# The COVID-19 health emergency: the influence of the Human Development Index on the reorganization of primary care

**DOI:** 10.3389/fpubh.2024.1440344

**Published:** 2024-11-28

**Authors:** Ana Paula de Vechi Corrêa, Ana Cristina Ribeiro, Rodrigo das Neves Cano, Gustavo Diego Magno, Carmen Álvarez-Nieto, Silvia Carla da Silva André Uehara

**Affiliations:** ^1^Nursing Department of Universidade Federal de São Carlos – UFSCar, São Carlos, Brazil; ^2^Nursing Department of Universidad de Jaén, Jaén, Spain

**Keywords:** COVID-19, development indicators, primary health care, public health surveillance, health management

## Abstract

**Objective:**

This study aimed to analyze and compare the organization and care offered by Primary Health Care (PHC) to people with suspected and/or diagnosed COVID-19 in Brazilian municipalities, considering the Human Development Index (HDI).

**Methods:**

This is an analytical cross-sectional study carried out with managers of PHC services in Brazilian municipalities. The data were collected using a self-administered questionnaire via Google Forms between April and September 2022 and were analyzed using prevalence ratios, using a Poisson regression model with a random effect and a 5% significance level.

**Results:**

The results indicated that municipalities with a low HDI were the most adherent to measures to reorganize PHC services during the health emergency phase of the pandemic when compared to municipalities with a medium, high, or very high HDI.

**Conclusion:**

This may be related to the better organization and structure of this level of care in the municipalities analyzed, pointing to the need for management that considers the population’s living conditions when dealing with public health emergencies.

## 1 Introduction

In the last three years, the COVID-19 pandemic has caused more than 6 million deaths and more than 676 million cases worldwide (1, 2). In Brazil, from the confirmation of the first case of the disease in February 2020 to the first million, only 115 days passed, from then on, every thirty days, on average, another million cases were confirmed, reaching the peak of cases in 2020 in mid-July and then being observed a decline in the number of cases until December where it presented a second peak of cases of the year (3). Until December 2023, the country accumulated more than 38 million cases and more than 700 thousand deaths by COVID-19 (4).

Brazil, with more than 210 million inhabitants, has the sixth largest population and fifth largest territory in the world, as well as one of the most diverse populations in sociodemographic terms, which is heterogeneously distributed throughout the country. In addition to health factors, territorial and socio-economic factors have also contributed to a heterogeneous profile of COVID-19 distribution in Brazilian regions (3).

Thus, during the critical phase of the pandemic, characteristics related to the territory, climate, population density, socioeconomic factors, as well as access to and availability of health resources also interfered in the spread of the disease in Brazil (5).

With regard to health resources, Brazil has the Unified Health System (SUS), a public health system that guarantees free access for the entire population at all levels of care, from Primary Health Care (PHC) to hospital services, including urgent and emergency services, epidemiological, health and environmental surveillance and pharmaceutical assistance (6).

With regard to the management of actions and services in the SUS, this takes place through the participation of the Union, represented by the Ministry of Health, which acts as the national manager and is responsible for formulating, standardizing, supervising, monitoring and evaluating policies and actions; the Federated Units (UF), represented by the state health secretariats, which participate in the formulation of health policies and actions; and the municipalities, through municipal health managers who are responsible for planning, organizing, controlling, evaluating and executing health actions and services at the local level (6).

However, although the country has a free public health system throughout Brazil, the socio-economic factors of the different regions may have influenced adherence to coping measures during the health emergency phase, as well as the capacity of health services to care for people infected with SARS-CoV-23.

Public health outcomes can be influenced by the Human Development Index (HDI), which is an integrated index of life expectancy, education and standard of living. An analysis that evaluated the correlation between the HDI and the epidemiological indicators of COVID-19 in different countries showed that the higher the HDI, the higher the cumulative incidence rate of cases, deaths and tests performed (7). On the other hand, a study carried out in the Northeast of Brazil pointed to a predominance of COVID-19 cases and deaths among the population living in neighborhoods with low and very low HDI (8).

In addition, although the virus does not distinguish between social classes, the conditions for treating the disease are not equal, and social inequality can be reflected in the provision of health services, which can directly influence the management of the disease and the quality of care provided to users during the health emergency phase. This means that the state has a greater presence in central areas, especially due to the greater diversity and presence of essential services, while in peripheral areas the proportion of these services on offer is scarce (9).

In this context, to meet the needs of socially vulnerable populations and at-risk groups, Primary Health Care (PHC) must act in health crisis scenarios, coordinating with municipal surveillance, as well as establishing the organization of flows in health services and essential actions to deal with the pandemic (3, 10).

However, although the World Health Organization (WHO) has declared the end of the public health emergency, the global spread of COVID-19 is still characterized as a pandemic, with new strains of SARS-CoV-2 circulating, which requires PHC to monitor territories to identify suspected cases, as well as monitoring confirmed cases of COVID-19 (10–12).

Thus, the literature has highlighted the role of PHC in health crises, such as that experienced in the COVID-19 pandemic, since factors intrinsically related to the territory, such as educational level, per-person income, precarious living conditions, access to health services, and other socioeconomic aspects can influence the population’s health-disease process, as well as the spread and coping with SARS-CoV-2 (3–9, 12–14).

However, it is still unclear how regional differences have influenced the reorganization of care offered to the population with COVID-19 in PHC during the health emergency. Therefore, this study aimed to analyze and compare the organization and care offered by PHC to people with suspected and/or diagnosed COVID-19 in Brazilian municipalities, considering the HDI.

## 2 Method

This is a descriptive observational cross-sectional study carried out in PHC in Brazil. The population consisted of managers of PHC services in Brazilian municipalities that had at least one confirmed case of COVID-19 between February 26, 2020 and June 30, 2021. Managers were municipal health secretaries, health coordinators, PHC coordinators and other PHC managers who worked at a central level and not directly in PHC services.

In Brazil, there are 5,570 municipalities (15) and each municipality has a manager responsible for PHC, so the population considered for this study was the 5,570 municipal PHC managers/coordinators. A sample calculation was carried out, considering one manager per municipality, using a standard error of 5.82%, assuming a prevalence of 50% and a 95% confidence coefficient. The sample was defined as 1,134 participants.

To participate in the study, the inclusion criteria were to have been a PHC manager in the municipality for at least 3 months during the COVID-19 health emergency phase and the exclusion criteria were those responsible who were on leave and/or vacation during the pandemic period.

Data was collected through a self-administered questionnaire by PHC service managers on Google Forms, from April to September 2022. For data collection, the researchers themselves constructed a questionnaire based on the Ministry of Health’s Protocol for the Clinical Management of Coronavirus (COVID-19) in PHC (16).

The survey instrument included the following variables: identification of suspected cases of Flu Syndrome and COVID-19; measures to prevent contagion in health units; stratification of the severity of Flu Syndrome; therapeutic management and home isolation of mild cases; early diagnosis and referral to urgent/emergency services or hospitals for severe cases; immediate notification; clinical monitoring; community prevention measures and support for active surveillance.

In order to reach the target audience, an e-mail survey was initially carried out of all the municipal health secretariats in the country, and the questionnaire was then sent to the study population via e-mail. The survey was also publicized by the National Council of Health Secretaries (CONASS) and the National Council of Municipal Health Secretaries (CONASEMS), which stressed the importance of municipal participation and forwarded the instrument to the municipal health secretariats. CONASEMS supporters also collaborated in publicizing the survey to the Regional Health Departments. Thus, once the number of participants estimated by the sample had been reached, data collection was completed.

For the analysis of the Human Development Index (HDI), the classification published in the 2014 Global Human Development Report (GHDR) was considered, namely: low human development below 0.550; medium human development between 0.550 and 0.699; high human development between 0.700 and 0.799; and very high human development above 0.800 (17).

Initially, the data was described using absolute and percentage frequencies (qualitative variables) and measures such as mean, standard deviation, minimum, median and maximum (quantitative variables). The Poisson regression model with a random effect was used to estimate prevalence ratios comparing HDI ranges (18). All the analyses were carried out using SAS 9.4 software and a 5% significance level was adopted.

The study was approved by the Ethics Committee of the Federal University of São Carlos, CAAE 52527521.8.0000.5504.

## 3 Results

A total of 1,134 managers from municipalities across the country took part in the survey, 40.4% (458) from the Southeast, 27.9% (316) from the Northeast, 18.6% (211) from the South, 8.5% (97) from the North and 4.6% (52) from the Midwest. As for the HDI assessment, 624 (55.0%) municipalities had an average HDI, 39.2% (444) a high HDI, 4.3% (49) a low HDI and 1.5% (17) a very high HDI. About the characteristics of the participants, 79.4% (900) were female and 20.6% (234) male, with an average age of 39.7 years.

The descriptive analysis highlights that most municipalities adopted measures recommended by the Ministry of Health to reorganize PHC during the critical phase of the pandemic, such as adaptations to PHC services and physical structure, in addition to reorganization of the flow of care and referral and counter-referral services (Table 1). Also noteworthy is the allocation of health professionals considered at risk for developing the severe form of COVID-19 to home office and notification of suspected cases of COVID-19 within 24 h by most municipalities (Table 1).

**Table 1:** Adoption of the measures recommended by the Ministry of Health for the reorganization of PHC during the COVID-19 pandemic, by Brazilian municipalities according to HDI (Brazil, 2022).

Variable	HDI			
	Low (*n* = 49)	Medium (*n* = 624)	High (*n* = 444)	Very high (*n* = 17)
Have PHC services been adapted to cope with COVID-19?				
No	3 (6.1%)	57 (9.1%)	30 (6.8%)	1 (5.9%)
Yes	46 (93.9%)	567 (90.9%)	414 (93.2%)	16 (94.1%)
Has the flow of PHC been restructured during the COVID-19 pandemic?				
No	3 (6.1%)	64 (10.3%)	28 (6.3%)	0 (0%)
Yes	46 (93.9%)	560 (89.7%)	416 (93.7%)	17 (100%)
Have PHC referral and counter-referral services within the HCN changed during the COVID-19 pandemic?				
No	13 (26.5%)	277 (44.4%)	156 (35.2%)	5 (29.4%)
Yes	36 (73.5%)	347 (55.6%)	287 (64.8%)	12 (70.6%)
Frequency Missing = 1				
Has the physical structure of PHC services been adapted to meet the needs of suspected and confirmed COVID-19 cases?				
No	16 (32.6%)	242 (38.8%)	141 (31.8%)	7 (41.2%)
Yes	33 (67.4%)	382 (61.2%)	303 (68.2%)	10 (58.8%)
Have healthcare workers considered to be at risk of developing the severe form of COVID-19 been allocated to the home office?				
No	1 (2.0%)	70 (11.2%)	58 (13.1%)	2 (11.8%)
Yes	48 (98%)	554 (88.8%)	386 (86.9%)	15 (88.2%)
Are suspected cases of COVID-19 reported by health professionals within 24 h of being received by health units?				
No	0 (0%)	5 (0.8%)	6 (1.35%)	0 (0%)
Yes	49 (100%)	619 (99.2%)	438 (98.65%)	17 (100%)
Are COVID-19 cases notified in the HCN previously assessed by PHC?				
No	3 (6.1%)	63 (10.1%)	61 (13.7%)	4 (23.5%)
Yes	46 (93.9%)	560 (89.9%)	383 (86.3%)	13 (76.5%)
Frequency Missing = 1				
Did PHC services implement specific protocols for dealing with COVID-19?				
No	4 (8.2%)	39 (6.2%)	25 (5.6%)	0 (0%)
Yes	45 (91.8%)	585 (93.8%)	419 (94.4%)	17 (100%)
Did PHC health professionals receive any training in caring for suspected cases of COVID-19 and managing users diagnosed with COVID-19?				
No	3 (6.1%)	64 (10.3%)	46 (10.4%)	0 (0%)
Yes	46 (93.9%)	560 (89.7%)	398 (89.6%)	17 (100%)
Did health professionals receive an adequate amount of personal protective equipment (PPE)?				
No	0 (0%)	10 (1.6%)	3 (0.7%)	0 (0%)
Yes	49 (100%)	614 (98.4%)	441 (99.3%)	17 (100%)
Did health professionals receive personal protective equipment (PPE) of adequate quality?				
No	0 (0%)	7 (1.1%)	6 (1.3%)	1 (5.9%)
Yes	49 (100%)	617 (98.9%)	438 (98.7%)	16 (94.1%)
Were users of the health unit referred to other points in the HCN when they had flu-like syndrome with saturation of less than 95% when breathing spontaneously, in addition to having any comorbidity at risk of worsening COVID-19?				
No	1 (2.0%)	14 (2.2%)	15 (3.4%)	0 (0%)
Yes	48 (98.0%)	610 (97.8%)	429 (96.6%)	17 (100%)
Has information technology been used for pre-clinical care, diagnosis, case follow-up and consultations?				
No	17 (34.7%)	218 (34.9%)	131 (29.5%)	4 (23.5%)
Yes	32 (65.3%)	406 (65.1%)	313 (70.5%)	13 (76.5%)
Did users over the age of 60, those with immunosuppression, the chronically ill, pregnant women, and women in the puerperium receive priority care when they came to the health unit reporting signs and symptoms of flu-like illness?				
No	0 (0%)	9 (1.4%)	14 (3.1%)	0 (0%)
Yes	49 (100%)	615 (98.6%)	430 (96.9%)	17 (100%)
Were users considered to be at risk, monitored by telephone every 24 h?				
No	4 (8.2%)	83 (13.3%)	68 (15.3%)	7 (41.2%)
Yes	45 (91.8%)	541 (86.7%)	376 (84.7%)	10 (58.8%)
Were users with flu-like symptoms monitored by telephone every 48 h?				
No	7 (14.3%)	83 (13.3%)	57 (12.8%)	5 (29.4%)
Yes	42 (85.7%)	541 (86.7%)	387 (87.2%)	12 (70.6%)

It is worth highlighting the implementation of specific protocols to deal with COVID-19 in PHC services, training of PHC professionals to care for suspected cases of COVID-19, management of users diagnosed with COVID-19, and availability of Personal Protective Equipment (PPE), which were actions identified in most participating municipalities. The results show that the use of information technologies for telecare, priority care for users considered at risk and monitoring every 24 h and 48 h were also carried out by most municipalities (Table 1).

Changes in PHC referral and counter-referral services within the Health Care Network (HCN) were 32% (CI: 1.06; 1.64) more prevalent in municipalities with a low HDI than those with a medium HDI. Also, in low HDI municipalities, the reassignment of health professionals considered to be at risk of developing the severe form to the home office was 10% (CI: 1.05; 1.16) more prevalent than in medium HDI municipalities and 13% (CI: 1.06; 1.19) more prevalent when compared to high HDI municipalities (Table 2).

**Table 2:** Adoption of measures recommended by the Ministry of Health for the reorganization of PHC during the COVID-19 pandemic, by Brazilian municipalities according to low, medium, high and very high HDI (Brazil, 2022).

Variable	Low *vs* Medium		Low *vs* High		Low *vs* Very high	
	PR (95% CI)	*p*-value	PR (95% CI)	*p*-value	PR (95% CI)	*p*-value
Have PHC services been adapted to deal with COVID-19?						
Yes	1.03 (0.93; 1.14)	0.53	1.01 (0.91; 1.11)	0.89	1 (0.86; 1.16)	0.97
Has the flow of PHC been restructured during the COVID-19 pandemic?						
Yes	1.05 (0.96; 1.14)	0.28	1 (0.93; 1.08)	0.96	0.94 (0.87; 1.01)	0.09
Have PHC referral and counter-referral services within the HCN changed during the COVID-19 pandemic?						
Yes	1.32 (1.06; 1.64)	0.01	1.13 (0.92; 1.39)	0.23	1.04 (0.73; 1.49)	0.83
Has the physical structure of PHC services been adapted to deal with suspected and confirmed cases of COVID-19?						
Yes	1.1 (0.86; 1.41)	0.45	0.99 (0.78; 1.25)	0.91	1.14 (0.73; 1.78)	0.55
Have healthcare workers considered to be at risk of developing the severe form of COVID-19 been allocated to the home office?						
Yes	1.1 (1.05; 1.16)	<0.01	1.13 (1.06; 1.19)	<0.01	1.11 (0.93; 1.33)	0.25
Are suspected cases of COVID-19 reported by health professionals within 24 h of being received by health units?						
Yes	1.01 (1; 1.02)	0.02	1.01 (1; 1.02)	0.01	1 (1; 1)	-
Are COVID-19 cases notified in the HCN previously assessed by PHC?						
Yes	1.04 (0.97; 1.13)	0.26	1.09 (1; 1.18)	0.04	1.23 (1.02; 1.48)	0.03
Did PHC services implement specific protocols for dealing with COVID-19?						
Yes	0.98 (0.89; 1.07)	0.66	0.97 (0.89; 1.07)	0.56	0.92 (0.84; 1)	0.06
Did PHC health professionals receive any training in caring for suspected cases of COVID-19 and managing users diagnosed with COVID-19?						
Yes	1.05 (0.97; 1.13)	0.27	1.05 (0.96; 1.14)	0.27	0.94 (0.87; 1.01)	0.10
Did health professionals receive an adequate amount of personal protective equipment (PPE)?						
Yes	1.02 (1.01; 1.03)	<0.01	1.01 (1; 1.01)	0.08	1 (1; 1)	-
Did health professionals receive personal protective equipment (PPE) of adequate quality?						
Yes	1.01 (1; 1.02)	<0.01	1.01 (1; 1.02)	0.01	1.06 (0.94; 1.2)	0.33
Were users of the health unit referred to other points in the HCN when they had flu-like syndrome with saturation of less than 95% when breathing spontaneously, in addition to having any comorbidity at risk of worsening COVID-19?						
Yes	1 (0.96; 1.04)	0.92	1.01 (0.97; 1.06)	0.53	0.98 (0.94; 1.02)	0.30
Did you use information technology for patients’ telemedicine in pre-clinical care, diagnosis, case follow-up, and consultations?						
Yes	1 (0.78; 1.29)	0.98	0.93 (0.72; 1.18)	0.54	0.85 (0.58; 1.26)	0.43
Did users over the age of 60, those with immunosuppression, the chronically ill, pregnant women, and women in the puerperium receive priority care when they came to the health unit reporting signs and symptoms of flu-like illness?						
Yes	1.01 (1.01; 1.02)	<0.01	1.03 (1.01; 1.05)	<0.01	1 (1; 1)	-
Were users considered to be at risk, monitored by telephone every 24 h?						
Yes	1.06 (0.97; 1.16)	0.22	1.08 (0.99; 1.19)	0.09	1.56 (1.08; 2.25)	0.02
Were users with flu-like symptoms monitored by telephone every 48 h?						
Yes	0.99 (0.85; 1.15)	0.88	0.98 (0.84; 1.15)	0.83	1.21 (0.88; 1.68)	0.24

COVID-19 cases notified in the HCN were previously assessed by PHC with a prevalence that was 9% (CI: 1; 1.18) higher in municipalities with a low HDI when compared to municipalities with a high HDI and 23% (CI: 1.02; 1.48) higher when compared to municipalities with a very high HDI (Table 2).

The availability of PPE to professionals in adequate quantity was 2% (CI: 1.01; 1.03) more prevalent in municipalities with a low HDI than in those with a medium HDI. Also, in municipalities with a low HDI, the availability of PPE of adequate quality to professionals was 1% (CI: 1; 1.01) more prevalent when compared to municipalities with medium and high HDI (Table 2).

Priority care for risk groups was 3% (CI: 1.01; 1.05) more prevalent in municipalities with a low HDI than in municipalities with a high HDI. When monitoring these at-risk users every 24 h by telephone was assessed, the prevalence was 56% (CI: 1.08; 2.25) higher in municipalities with a low HDI when compared to municipalities with a very high HDI (Table 2).

When comparing PHC services in medium, high, and very high HDI municipalities, it was found that adapting these services to deal with COVID-19 was 10% less prevalent in medium HDI municipalities and 6% less prevalent in high HDI municipalities when compared to very high HDI municipalities (Table 3).

**Table 3:** Adoption of measures recommended by the Ministry of Health for the reorganization of PHC during the COVID-19 pandemic, by Brazilian municipalities according to medium, high and very high HDI (Brazil, 2022).

Variable	Medium *vs* High		Medium *vs* Very high		High *vs* Very high	
	PR (95% CI)	*p*-value	PR (95% CI)	*p*-value	PR (95% CI)	*p*-value
Have PHC services been adapted to deal with COVID-19?						
Yes	0.97 (0.93; 1.02)	0.28	0.97 (0.85; 1.09)	0.57	0.99 (0.88; 1.12)	0.88
Has the flow of PHC been restructured during the COVID-19 pandemic?						
Yes	0.96 (0.92; 1)	0.06	0.9 (0.87; 0.93)	<0.01	0.94 (0.91; 0.96)	<0.01
Have PHC referral and counter-referral services within the HCN changed during the COVID-19 pandemic?						
Yes	0.86 (0.76; 0.97)	0.01	0.79 (0.57; 1.09)	0.14	0.92 (0.67; 1.26)	0.59
Frequency Missing = 1						
Has the physical structure of PHC services been adapted to deal with suspected and confirmed cases of COVID-19?						
Yes	0.9 (0.81; 1)	0.05	1.04 (0.71; 1.53)	0.84	1.16 (0.79; 1.71)	0.45
Have healthcare workers considered to be at risk of developing the severe form of COVID-19 been allocated to the home office?						
Yes	1.02 (0.97; 1.07)	0.41	1.01 (0.84; 1.2)	0.95	0.99 (0.82; 1.18)	0.87
Are suspected cases of COVID-19 reported by health professionals within 24 h of being received by health units?						
Yes	1.01 (0.99; 1.02)	0.40	0.99 (0.99; 1)	0.02	0.99 (0.98; 1)	0.01
Are COVID-19 cases notified in the HCN previously assessed by PHC?						
Yes	1.04 (0.99; 1.09)	0.09	1.18 (0.98; 1.4)	0.07	1.13 (0.94; 1.35)	0.19
Frequency Missing =1						
Did PHC services implement specific protocols for dealing with COVID-19?						
Yes	0.99 (0.96; 1.02)	0.68	0.94 (0.92; 0.96)	<0.01	0.94 (0.92; 0.97)	<0.01
Did PHC health professionals receive any training in caring for suspected cases of COVID-19 and managing users diagnosed with COVID-19?						
Yes	1 (0.96; 1.05)	0.96	0.9 (0.87; 0.92)	<0.01	0.9 (0.87; 0.93)	<0.01
Did health professionals receive an adequate amount of personal protective equipment (PPE)?						
Yes	0.99 (0.98; 1)	0.16	0.98 (0.97; 0.99)	<0.01	0.99 (0.99; 1)	0.08
Did health professionals receive personal protective equipment (PPE) of adequate quality?						
Yes	1 (0.99; 1.02)	0.74	1.05 (0.93; 1.19)	0.43	1.05 (0.93; 1.19)	0.46
Were users of the health unit referred to other points in the HCN when they had flu-like syndrome with saturation of less than 95% when breathing spontaneously, in addition to having any comorbidity at risk of worsening COVID-19?						
Yes	1.01 (0.99; 1.03)	0.29	0.98 (0.97; 0.99)	<0.01	0.97 (0.95; 0.98)	<0.01
Did you use information technology for patients’ telemedicine in pre-clinical care, diagnosis, case follow-up, and consultations?						
Yes	0.92 (0.83; 1.02)	0.13	0.85 (0.62; 1.17)	0.33	0.92 (0.67; 1.27)	0.62
Did users over the age of 60, those with immunosuppression, the chronically ill, pregnant women, and women in the puerperium receive priority care when they came to the health unit reporting signs and symptoms of flu-like illness?						
Yes	1.02 (1; 1.04)	0.10	0.99 (0.98; 1)	<0.01	0.97 (0.95; 0.99)	<0.01
Were users considered to be at risk, monitored by telephone every 24 h?						
Yes	1.02 (0.96; 1.09)	0.45	1.47 (1.03; 2.11)	0.03	1.44 (1.01; 2.06)	0.05
Were users with flu-like symptoms monitored by telephone every 48 h?						
Yes	0.99 (0.94; 1.05)	0.85	1.23 (0.92; 1.64)	0.17	1.23 (0.92; 1.65)	0.16

Changes in PHC referral and counter-referral services within the HCN during the COVID-19 pandemic were 14% (IC: 0.76; 0.97) less prevalent in municipalities with a medium HDI compared to municipalities with a high HDI. Notification of suspected COVID-19 cases within 24 h was 1% (CI: 0.98; 1) less prevalent in municipalities with medium and high HDI when compared to municipalities with very high HDI (Table 3).

The implementation of specific protocols for dealing with COVID-19 was 6% (CI: 0.92; 0.96) less prevalent in medium and high HDI municipalities when compared to very high HDI municipalities. Training of PHC health professionals in the management of suspected or confirmed COVID-19 cases was 10% (CI: 0.87; 0.93) less prevalent in medium and high HDI municipalities when compared to very high HDI municipalities (Table 3).

The availability of PPE to professionals in adequate quantities was 2% (CI: 0.97; 0.99) less prevalent in municipalities with a medium HDI than in those with a very high HDI. In addition, in terms of referring patients at risk of worsening COVID-19 to other points in the HCN, the prevalence was 2% (CI: 0.97; 0.99) lower in municipalities with a medium HDI and 3% (0.95; 0.98) lower in municipalities with a high HDI when compared to municipalities with a very high HDI (Table 3).

Priority care for risk groups was 1% (CI: 0.98; 1) less prevalent in medium HDI municipalities and 3% (CI: 0.95; 0.99) less prevalent in high HDI municipalities than in those with very high HDI. When monitoring these at-risk users every 24 h by telephone was assessed, the prevalence was 47% (CI: 1.03; 2.11) higher in municipalities with a high HDI when compared to those with a very high HDI (Table 3).

## 4 Discussion

Analysis of the results indicates that PHC services were reorganized to deal with the high demand for COVID-19 cases during the critical phase of the pandemic; however, this reorganization took place at different times and with different quality among Brazilian municipalities. It should be noted that the lack of national guidelines had an impact on the process of reorganizing services, especially PHC, as well as the characteristics and indicators of the municipalities, such as the HDI.

The non-pharmacological measures adopted to reduce the spread of COVID-19 and the management of mild cases of the disease should have been widely disseminated and carried out by PHC services, but the emphasis on hospital care has led to fragmentation in the reorganization of PHC to cope with the pandemic. PHC services have key characteristics for dealing with outbreaks and epidemics, such as knowledge of the territory, access, a link between the user and the health team, comprehensive care, and monitoring of vulnerable families (19).

The lack of national coordination and even government cooperation between the federal and state governments, coupled with the scarcity and slowness in allocating resources and adopting measures to reorganize the health system, directly affected the quality of the reorganization of PHC services during the critical phase of the pandemic (20).

Throughout these years of the pandemic, one of the main lessons was that dealing with a health emergency requires national technical coordination, as well as the reorganization of health services, combining individual care with community-centered care. Thus, the experience during the critical phase of the COVID-19 pandemic has highlighted the importance and effectiveness of health systems strongly anchored in PHC, so they can offer comprehensive, continuous, and articulate care, responding better to health emergencies (21, 22).

PHC services are the preferred access to the health system, the place of reference where people should seek care (18). However, during physical isolation in the critical phase of the pandemic, this access was weakened, as many services were interrupted, which also had repercussions on referral services (23). In this scenario, it is considered that municipalities with low HDI offered limited access to specialized and hospital services, making PHC the main reference in health care, in addition to requiring effective referral and counter-referral services to ensure equitable health care.

Although it is important to prioritize the coverage of PHC services, especially the Family Health Strategy (FHS), there is a caveat that greater coverage alone does not meet the health needs of the most vulnerable populations, but the inclusion of actions relating to the work process, availability of inputs, definition of flows in the Health Care Network (HCN), facilitated access to PHC and other levels of care and equity so that more quality can be achieved in the production of care (24).

The marked social, economic, and access to health services inequalities observed in many Brazilian municipalities came to light during the COVID-19 health emergency (25, 26). However, contrary to what was expected, the greater availability and better quality of PPE in municipalities with lower HDI may be related to tripartite management, i.e., the responsibility of the Union, states, and municipalities for health management, thus the distribution of PPE in the COVID-19 health emergency was carried out by the three federated entities and not only by the municipalities (27).

However, there was a rush by countries to acquire PPE, with most of this equipment going to hospitals and as the pandemic progressed in Brazil, access to PPE for health professionals became a concern, since PPE shortages were occurring in most countries (28). One of the legacies of the COVID-19 pandemic refers to the importance of maintaining PPE in health services being considered a state policy, to foster the national industry and guarantee production in adequate quantity and quality to deal with a health emergency.

During the critical phase of the pandemic, the higher prevalence of care for people considered to be in the risk group for developing the severe form of COVID-19 in municipalities with lower HDI may be justified by the wide access to PHC services by the population in these municipalities, since a small portion of the population has access to private health services, unlike what happens in municipalities with higher HDI.

To justify this inference, the pre-pandemic period should be analyzed, in which the expansion of the Family Health Strategy (FHS) occurred mostly in inland municipalities and in areas of metropolitan regions where people with lower socioeconomic status live. Thus, in 2017, FHS coverage was 70% in inland municipalities and 45.5% in state capitals, directly implying access to health insurance by lower-income families (29).

Less vulnerable municipalities with a higher HDI had more cases and more deaths from COVID-19, and municipalities with greater social vulnerability and lower HDI had fewer diagnostic tests, which may have influenced the number of cases identified (30).

On the international scene, an analysis carried out in Lima (Peru) found no relationship between mortality and lethality coefficients from COVID-19 and HDI; however, there was an association between population density and these indicators (31). The organization and availability of financial and human resources do not imply the spread of COVID-19, but are directly related to the measures implemented to diagnose cases, effective notification, and access to health services and testing, so these municipalities tend to have a high number of cases, but not necessarily deaths, since the structuring of the health care network has a direct impact on the outcome of cases.

In this context, the richest areas of the city of Rio de Janeiro had a high infection rate, but a low fatality rate; on the other hand, a large portion of the more impoverished neighborhoods with the population most exposed to social vulnerabilities had the highest mortality rate (9).

Based on the results found, it can be inferred that the wealthier the municipality, the fewer adaptations were made to PHC services. In wealthier municipalities, the public health system was also prevalent in COVID-19 care, but a considerable part of the population, with better purchasing power, sought private services more often. On the other hand, it should be noted that many municipalities with higher HDI are also points in the HCN, especially with regard to the hospital sector, directly impacting on the restructuring of services within the network, from the moment cases are notified.

Another relationship between the three federal entities is configured in the supply and spatialization of health services, with the state having a greater presence in prime and central areas, offering a wider range of essential services; however, in peripheral areas, the availability and access to these services is less and more difficult. In this way, a logic of social inequality is configured, evidenced by the quantitative and qualitative concentration of services in the urban space, as well as their access (9).

In regions of high vulnerability, the supply of highly complex health services is dependent on the private network contracted to the SUS, which is a limitation in the management of comprehensive care (32). Also, noteworthy are the Inter-Municipal Health Consortia, which are strategies for providing the population with specialized and highly complex health services (33).

The socioeconomic status of the municipality has a direct impact on the organization and availability of health services. It can be seen that municipalities with the best HDI have implemented protocols for the management of COVID-19 in PHC and have trained professionals more frequently. Thus, we understand the direct relationship between economic, educational, and managerial indicators in the efficiency of health care offered to users, as well as in the ability to reorganize health services efficiently and skillfully to deal with a health emergency (34).

Analysis of the evolution of the pandemic allows us to infer that the health system’s ability to save lives was not only related to the number of ICU beds and ventilators but also the reorganization of the HCN, to guarantee easy access and assertive care in any health service. The lack of efficient organization in the country has emerged from the shortcomings of the HCNs, most of which are fragile and fragmented, as well as limited and in many places inefficient PHCs. This situation has been caused recently by the lack of public policies aimed at strengthening PHC to guarantee coordination, continuity, and comprehensive care (35).

Thus, this study indicated that the municipalities with the lowest HDI had a more adequate reorganization of PHC services during the COVID-19 health emergency phase, a fact that may be related to better organization and structure at this level of care in these locations. In addition, it was possible to identify that PHC services were organized in different ways in the municipalities, demonstrating the direct interference of individual characteristics of the municipalities such as HDI.

Historically, the influence of socioeconomic conditions on disease morbidity and mortality has been well known, pointing to the need for management that considers the population’s living conditions when dealing with public health emergencies.

One of the limitations of this study was the low level of participation by managers in the North and Midwest regions, as well as the Brazilian capitals, and it is important to mention that the sample is not representative of all regions of the country. However, even in the face of low adherence in some regions, managers from all Brazilian states took part in the study and the results present important analyses that can serve as input for planning policies to deal with health emergencies in PHC services, considering the differences between municipalities.

This study provides input for planning policies to deal with health emergencies in PHC services, taking into account the differences between municipalities. The knowledge generated in this study contributes to implementing and strengthening public health policies that consider the population’s living conditions when dealing with other public health emergencies.

## Data Availability

The raw data supporting the conclusions of this article will be made available by the authors, without undue reservation.
